# Long‐term bowel function following delayed coloanal anastomosis: Analysis of a multicentric cohort study (GRECCAR)

**DOI:** 10.1111/codi.70013

**Published:** 2025-02-04

**Authors:** Maxime K. Collard, Jean‐Jacques Tuech, Charles Sabbagh, Amine Souadka, Jérome Loriau, Eric Rullier, Frédéric Marchal, Adeline Germain, Stéphane Benoist, Jean‐Luc Faucheron, Gilles Manceau, Anne Dubois, Anaïs Laforest, Isabelle Sourrouille, Aurore Protat, Diane Mège, Zaher Lakkis, Michel Prudhomme, Simon Derieux, Mehdi Ouaissi, Aurélien Venara, Cécile Brigand, Bernard Lelong, Karine Pautrat, Leon Maggiori, Gil Lebreton, Philippe Rouanet, Marc Pocard, Emilie Duchalais, Quentin Denost, Yann Parc, Jérémie H. Lefevre, for the GRECCAR Group

**Affiliations:** ^1^ Department of Colorectal Surgery, Hôpital Saint‐Antoine, Assistance Publique Hôpitaux de Paris Sorbonne University Paris France; ^2^ Department of General and Digestive Surgery Hôpital Charles Nicole Rouen France; ^3^ Department of General and Digestive Surgery Hôpital d'Amiens Amiens France; ^4^ Department of General and Digestive Surgery National Institute of Oncology Rabat Morocco; ^5^ Department of General and Digestive Surgery Hôpital Saint‐Joseph Paris France; ^6^ Department of General and Digestive Surgery Saint André Hospital Bordeaux France; ^7^ Department of General and Digestive Surgery Institut de cancérologie de Lorraine Vandoeuvre‐les‐Nancy France; ^8^ Department of General and Digestive Surgery Hôpital Universitaire de Nancy Nancy France; ^9^ Department of General and Digestive Surgery Hôpital du Kremlin‐Bicêtre Kremlin‐Bicêtre France; ^10^ Department of Colorectal Surgery Hôpital Unversitaire de Grenoble La Tronche France; ^11^ Department of General and Digestive Surgery Hôpital Européen Georges Pompidou Paris France; ^12^ Department of General and Digestive Surgery CHU Clermont‐Ferrand Site Estaing Clermont‐Ferrand France; ^13^ Department of General and Digestive Surgery Institut Monsouris Paris France; ^14^ Gustave Roussy, Département d'Anesthésie Chirurgie et Interventionnel Villejuif France; ^15^ Department of General and Digestive Surgery Hôpital Huriez Lille France; ^16^ Department of General and Digestive Surgery Hôpital de la Timone Marseille France; ^17^ Department of Digestive Surgery University Hospital of Besancon Besancon France; ^18^ Department of General and Digestive Surgery Hôpital Universitaire de Nîmes Nîmes France; ^19^ Department of General and Digestive Surgery Groupe Hospitalier Diaconesses – Croix Saint Simon Paris France; ^20^ Department of General and Digestive Surgery Hôpital Trousseau – CHRU Hôpitaux de Tours Chambray‐lès‐Tours France; ^21^ Department of General and Digestive Surgery Hôpital Universitaire d'Angers Angers France; ^22^ Department of General and Digestive Surgery Hôpital de Hautepierre – Hôpitaux Universitaires Strasbourg France; ^23^ Department of General and Digestive Surgery Institut Paoli‐Calmettes Marseille France; ^24^ Department of General and Digestive Surgery Hôpital Lariboisière Paris France; ^25^ Department of General and Digestive Surgery CHU côte de Nâcre Caen France; ^26^ Department of General and Digestive Surgery Institut du Cancer de Montpellier Montpellier France; ^27^ Department of General and Digestive Surgery Hôpital Pitié‐Salpêtrière Paris France; ^28^ Department of General and Digestive Surgery Centre Hospitalier Universitaire de Nantes Nantes France; ^29^ Department of General and Digestive Surgery Bordeaux Colorectal Institute Bordeaux France

**Keywords:** anterior resection syndrome, coloanal anastomosis, LARS, rectal cancer

## Abstract

**Aim:**

Alteration of bowel function after delayed coloanal anastomosis (DCAA) might be a limitation to its utilization. Our aim was to assess the long‐term bowel function of DCAA in a large multicentric cohort.

**Method:**

All patients who underwent DCAA interventions at 29 GRECCAR‐affiliated hospitals between 2010 and 2021 were retrospectively included. Low anterior resection syndrome (LARS) score or confection of a stoma due to poor bowel function was assessed in eligible patients. Good bowel function was defined by the preservation of bowel continuity with no LARS or a minor LARS.

**Results:**

Among the 385 eligible patients to assess long‐term bowel continuity, 63% (*n* = 243) responded to the questionnaire or had a definitive stoma because of poor bowel function. After a median follow‐up of 32 months, good bowel function was reported by 60% (*n* = 146) of patients (with no LARS 36% and minor LARS 24%), whereas 40% of patients (*n* = 146) had a poor bowel function including major LARS (36%) and definitive stoma due to poor bowel function (4%). No variables tested were predictive of a poor bowel function after DCAA, including a history of pelvic radiotherapy (*P* = 0.722), salvage DCAA after failure of a previous anastomosis (*P* = 0.755), presence of a diverting stoma (*P* = 0.556), occurrence of an anastomotic leakage (*P* = 0.416) and time interval from the DCAA to the bowel function assessment (*P* = 0.350).

**Conclusions:**

No LARS or minor LARS was reached for 60% of patients after DCAA. Less than 5% of patients received a definitive stoma due to a poor bowel function.

**What does this paper add to the literature?**

This paper evaluates bowel function after delayed coloanal anastomosis in a multicentre cohort representative of practice diversity. The study finds bowel function comparable to that reported for immediate coloanal anastomosis in the literature, implying that the utilization of this technique should not be constrained by concerns regarding anticipated bowel function.

## INTRODUCTION

Coloanal anastomosis (CAA) with a diverting stoma is usually performed concomitantly with rectal resection and mobilization of the left colon, corresponding to an immediate coloanal anastomosis (ICAA) [1]. An alternative option, known as delayed coloanal anastomosis (DCAA) [2, 3], consists of pulling a stump of colon through the anus and leaving it protruding in this position for a period of 1–4 weeks without performing the anastomosis [4, 5, 6]. Subsequently, the colonic stump is sectioned and the CAA is handsewn. The objective of this time interval is to encourage the formation of scar tissue adhesions between the colon and the anus before fashioning the anastomosis, with the aim of reducing the occurrence of anastomotic leakage (AL) [6]. This technique serves two indications. The first is when DCAA is performed without a diverting stoma (DCAA‐NoStoma) as an alternative to ICAA protected by a diverting stoma [2, 4, 7] in order to avoid stoma‐related complications for the patients [8, 9]. Second, DCAA can be performed in addition to a diverting stoma (DCAA‐Stoma) in patients at very high risk of AL, especially in cases of redo anastomosis after the failure of a previous colorectal anastomosis (CRA) or redo CAA [10, 11] or in the case of chronic rectovaginal [12] or rectourethral fistula [13].

The specific impact of DCAA on bowel function is not well understood and has been reported either in the context of DCAA‐NoStoma [4, 7] or in the diametrically opposed context of DCAA‐Stoma [14] without ever considering all the indications of this technique simultaneously. It is crucial to elucidate the functional outcome of this surgical intervention since there is suspicion among some that leaving the colonic stump through the anal canal for several days before the confection of the anastomosis could adversely affect long‐term anal sphincter continence. Moreover, factors potentially influencing the bowel function after DCAA, such as the time interval between the two surgical steps, are unexplored.

Our aim was to evaluate the long‐term bowel function with the risk of a low anterior resection syndrome (LARS) following DCAA within a large multicentric cohort, including all the indications. The secondary objective was to explore modifiable and non‐modifiable risk factors that could impact the bowel function in this context.

## METHODS

### Patients

All patients who underwent a DCAA between January 2010 and June 2021 at 30 affiliated tertiary referral colorectal centres associated with the French Research Group of Rectal Cancer Surgery (GRECCAR) were retrospectively included in the DCAA cohort. Twenty‐nine out of 30 participating centres agreed to take part in this study, which focused on bowel function and involved a specific questionnaire that had to be sent to all eligible patients. As the main objective of our study was to assess the long‐term bowel function after DCAA, we excluded (1) patients who had died before being contacted for the assessment of bowel function, (2) patients without restoration of bowel continuity after DCAA, (3) patients with a permanent stoma due to an AL or a pelvic tumour recurrence and (4) patients with bowel continuity in the latest follow‐up who did not respond to the questionnaire assessing bowel function. Finally, patients included in the current study were patients with preserved digestive continuity at the last follow‐up who responded to the questionnaire on bowel function and patients who decided to have a definitive stoma performed due to poor functional results of the DCAA.

### Surgical procedure: DCAA

The surgical procedure for DCAA employed by the participating centres was thoroughly detailed in the previous GRECCAR study on DCAA [5]. Briefly, the principle of DCAA is to perform a CAA in two steps. The first step consists of anterior resection of the rectum or resection of the previous CRA or CAA and the colon is then pulled through the anus, covering a length of 5–10 cm. In the case of insufficient colonic length, the colon is descended through the mesentery (Toupet procedure) or the inverted right colon is used (Deloyers’ procedure) [15, 16, 17, 18]. DCAA is protected by a diverting stoma (DCAA‐Stoma) when the aim of the DCAA is to optimize the anastomotic healing in patients with a high risk of AL, whereas DCAA‐NoStoma is performed when the aim is to avoid a stoma for a CAA, as an alternative to the ICAA protected by a diverting stoma. The time interval between the two surgical stages is at the discretion of the operating surgeon. The second step of DCAA relies on the section of the exteriorized colonic stump, followed by a direct single‐layer handsewn CAA using interrupted absorbable sutures. For DCAA‐Stoma patients, the reversal of the diverting stoma is scheduled 6–8 weeks after DCAA, when no AL is diagnosed following a CT scan with water‐soluble contrast administered through the stoma.

### Data collection and bowel function assessment

All data, except information about the bowel function, were retrospectively collected by a designated surgeon in each centre. To assess the bowel function after DCAA, all patients eligible for its evaluation were contacted by the treating surgeons and by phone, mail or during a consultation in clinics and, after obtaining consent, they were asked to answer the LARS score, which is a standardized score assessing specifically the bowel function after rectal resection [19]. This score consists of five questions, each with three to four possible answers. The overall score determines the value of the LARS score, ranging from 0 to 42. This score is categorized into three levels: no LARS (score between 0 and 20), minor LARS (score between 21 and 29) and major LARS (score between 30 and 42). Good bowel function was defined by patients with bowel continuity preserved after DCAA with no LARS or with minor LARS (LARS score < 30), whereas poor bowel function was considered for patients presenting major LARS or patients with a definitive stoma fashioned because of poor bowel function.

### Definition of variables and outcomes

Overall postoperative morbidity was considered as any deviation from the normal postoperative course that occurred from the day of the first stage of DCAA until 90 days after the second step of DCAA. Morbidity was graded according to the Clavien–Dindo classification [20]. A complication classified as Clavien–Dindo III or higher was considered as major. AL was defined as a communication between the intraluminal and extraluminal compartments due to a defect of the integrity of the CAA. Any pelvic abscess that occurred after the formation of the CAA was also considered as AL [21]. A pelvic abscess diagnosed after the first step of DCAA but before the construction of the anastomosis was not considered as an AL.

### Statistical analysis

Qualitative data were reported as frequencies and percentages and two‐group comparison was performed with the *χ* ^2^ test for expected cell counts ≥5 and with the Fisher exact test if not. Quantitative data were expressed as medians and interquartile range (IQR) and compared between two groups using the Mann–Whitney *U* test. This test was also used for the two‐group comparison of ordinal data. All tests were two‐sided. A *P* value of <0.05 was considered statistically significant. All analyses were performed using the R Software version 4.2.3 (R Core Team, Austria).

This study was conducted according to the ethical standards of the Committee on Human Experimentation of each institution and reported according to the Strengthening the Reporting of Observational Studies in Epidemiology (STROBE) guidelines [22]. This study was approved by the Research Ethics Committee of the Rouen University Hospital (reference number E2024‐76).

## RESULTS

### Population

**FIGURE 1:**
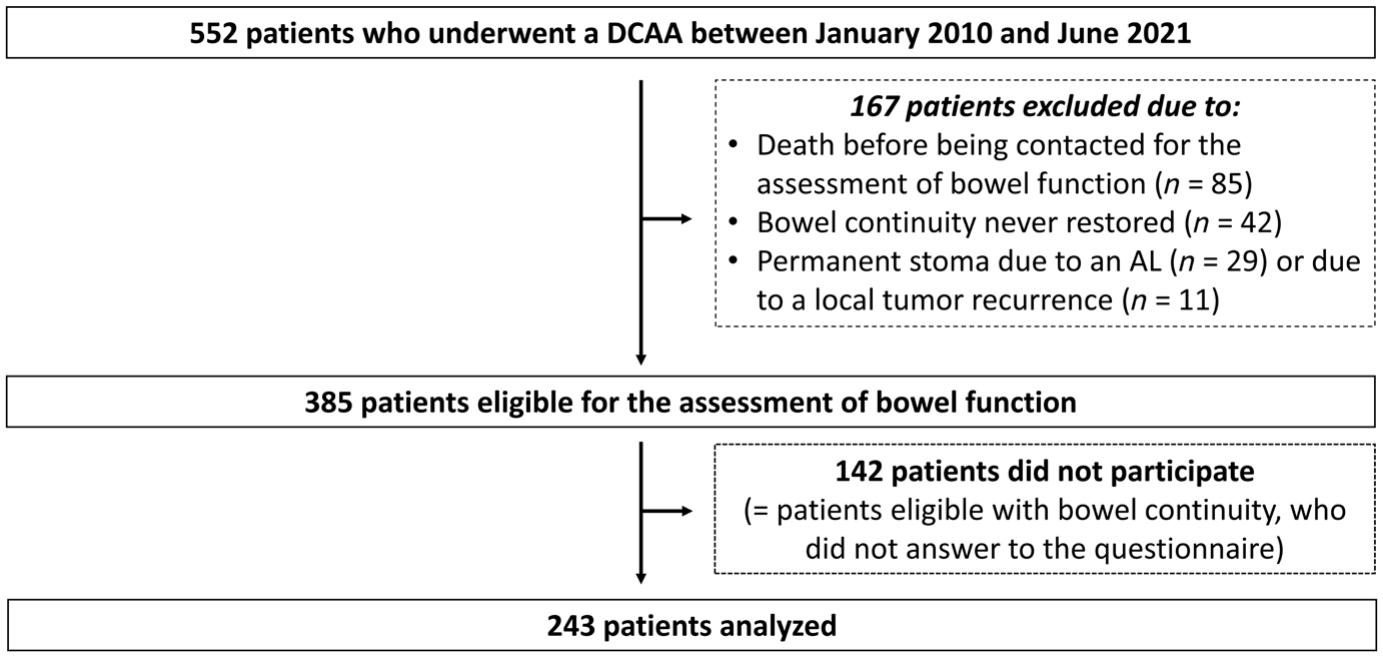
Flow chart of the study.

Among the 552 patients who underwent DCAA in the 29 participating centres, 85 patients died before being contacted for the assessment of bowel function, 42 never had bowel continuity restored and 40 had a permanent stoma secondarily fashioned due to a pelvic tumour recurrence (*n* = 11) or to an AL (*n* = 29). Among eligible patients to assess long‐term bowel continuity (*n* = 385), 63% (*n* = 243) responded to the questionnaire or had a definitive stoma because of poor bowel function (Figure 1). The comparison between the responder and non‐responder populations is presented in Table 1. Notably, there were significantly more patients who underwent redo anastomosis (history of CRA/CAA) (*P* < 0.001) and those who had surgery with a diverting stoma (*P* < 0.001) in the responder group.

**TABLE 1:** Characteristics of eligible patients to answer to the LARS score and of responder and non‐responder patients to this score.

Variables	Eligible patients (*n* = 385)	Non‐responders (*n* = 142)	Responders (*n* = 243)	*P* value	Missing value: Eligible/no responders/responders
Preoperative characteristics					
Female	156 (41)^a^	50 (35)	106 (44)	0.105	0/0/0
Age (years)	62.4 (IQR 53.1–69.4)^b^	62.9 (IQR 56.5–69.0)^b^	61.9 (IQR 51.3–70.1)	0.172	1/1/0
BMI (kg/m^2^)	24.9 (IQR 22.2–28.3)	25.6 (IQR 22.8–29.1)	24.6 (IQR 21.9–27.2)	0.009	12/6/6
ASA score ≥ 3	79 (21)	26 (18)	53 (22)	0.431	1/1/0
Active smoker	63 (23)	21 (23)	42 (22)	0.836	106/52/54
Antiplatelet therapy	41 (12)	13 (13)	28 (12)	0.917	56/40/16
Anticoagulant therapy	21 (7.0)	6 (6.1)	15 (7.5)	0.647	86/43/43
Diabetes	37 (11)	11 (11)	26 (11)	>0.999	62/46/16
Previous pelvic radiotherapy	260 (68)	94 (66)	166 (68)	0.669	0/0/0
Primary disease				0.067	0/0/0
Colorectal cancer	337 (88)	124 (87)	213 (88)		
Endometriosis	16 (4.2)	3 (2.1)	13 (5.3)		
Gynaecological/urological malignancy	8 (2.1)	6 (4.2)	2 (0.8)		
Epidermoid carcinoma of the anus	3 (0.8)	2 (1.4)	1 (0.4)		
Others	21 (5.5)	7 (4.9)	14 (5.8)		
Surgical procedure					
Redo anastomosis (failure of previous CRA/CAA)	106 (28)	23 (16)	83 (34)	<0.001	0/0/0
Redo anastomosis (history of CRA/CAA) in the early postoperative period (≤21 days)	20 (5.2)	2 (1.4)	18 (7.4)	0.010	0/0/0
Surgical approach (first step of DCAA)				<0.001	1/1/0
Laparotomy	195 (51)	72 (51)	123 (51)		
Laparoscopy	152 (40)	41 (29)	111 (46)		
Robot‐assisted	37 (9.6)	28 (20)	9 (3.7)		
Toupet procedure	28 (7.3)	5 (3.5)	23 (9.5)	0.030	0/0/0
Deloyers' manoeuvre	12 (3.1)	3 (2.1)	9 (3.7)	0.547	0/0/0
	105 (27)	22 (15)	83 (34)	<0.001	0/0/0
Time interval between two steps of DCAA (days)	7.0 (IQR 6.0–10.0)	7.0 (IQR 5.0–9.0)	8.0 (IQR 7.0–11.0)	<0.001	15/7/8
Postoperative course					
Overall morbidity	189 (49)	69 (49)	120 (49)	0.881	0/0/0
Major morbidity	78 (20)	32 (23)	46 (19)	0.396	0/0/0
Intra‐abdominal colon necrosis	19 (4.9)	7 (4.9)	12 (4.9)	0.997	0/0/0
Necrosis of the exteriorised colon	8 (2.1)	4 (2.8)	4 (1.6)	0.474	0/0/0
Pelvic abscess between two steps of DCAA	20 (5.2)	8 (5.6)	12 (4.9)	0.767	0/0/0
Anastomotic leakage (AL)	34 (8.8)	11 (7.7)	23 (9.5)	0.566	0/0/0
Length of stay (days)	14.0 (IQR 11.0–19.0)	13.0 (IQR 9.0–17.0)	14.5 (IQR 12.0–20.0)	<0.001	20/11/9

Abbreviations: ASA score, American Society of Anesthesiologists score; BMI, body mass index; CAA, coloanal anastomosis; CRA, colorectal anastomosis; DCAA, delayed coloanal anastomosis; IQR, interquartile range.

^a^ *n*/*N* (%).

^b^ Median IQR (25%–75%).

The median age of the 243 responder patients was 61.9 years, and 44% of the patients were women. The primary disease leading to colorectal surgery was predominantly colorectal cancer (213/243, 88%). Sixty‐eight per cent of the patients received pelvic radiotherapy before the surgical procedure. The DCAA followed the failure of a previous CRA or CAA in 34% of cases (83/243). No patient in this cohort had an intersphincteric resection associated with DCAA. The DCAA was performed without a diverting stoma (DCAA‐NoStoma) for 66% of patients (160/243). The overall morbidity and severe morbidity were 49% and 19%, respectively. The rate of AL was 9.5%. Details on the patient characteristics are reported in Table 1.

### LARS score and functional outcomes

The median time interval between the DCAA procedure and the assessment of bowel function was 32.0 months (IQR 17.7–50.0). Good bowel function with preservation of digestive continuity after DCAA was achieved in 60% of patients (146/243), including 36% of patients with no LARS and 24% of patients with minor LARS. Among the 40% (87/243) of patients who experienced poor bowel function, 36% had preservation of digestive continuity with major LARS and 4% received a secondary definitive stoma due to poor bowel function after the DCAA. Details regarding the functional outcome obtained in the cohort are presented in Table 2.

**TABLE 2:** Details of bowel function after DCAA.

Variables	Patients
Bowel function	
Bowel continuity with no LARS	87/243 (36)^a^
Bowel continuity with minor LARS	59/243 (24)
Bowel continuity with major LARS	88/243 (36)
Stoma due to poor bowel function	9/243 (3.7)
LARS score^b^	26.0 (IQR 15.0–34.0)^c^
Bowel function details^d^	
Daytime faecal frequency	
<1 per day: 4 points	25/187 (13%)
Between 1 and 3 per day: 2 points	66/187 (35%)
Between 4 and 7 per day: 0 point	83/187 (44%)
>7 per day: 5 points	13/187 (7.0%)
Gas incontinence	143/187 (76%)
Leakage of liquid stool	132/187 (71%)
Faecal fragmentation	143/187 (76%)
Faecal urgencies	124/187 (66%)

Abbreviations: DCAA, delayed coloanal anastomosis; IQR, interquartile range; LARS, low anterior resection syndrome.

^a^ *n*/*N* (%).

^b^ Among patients with bowel continuity (no LARS score in patients with a stoma due to a poor function result).

^c^ Median IQR (25%–75%).

^d^ Among patients with bowel continuity (exclusion of patients without details given about the LARS score).

### Risk factors of poor bowel function after DCAA

**FIGURE 2:**
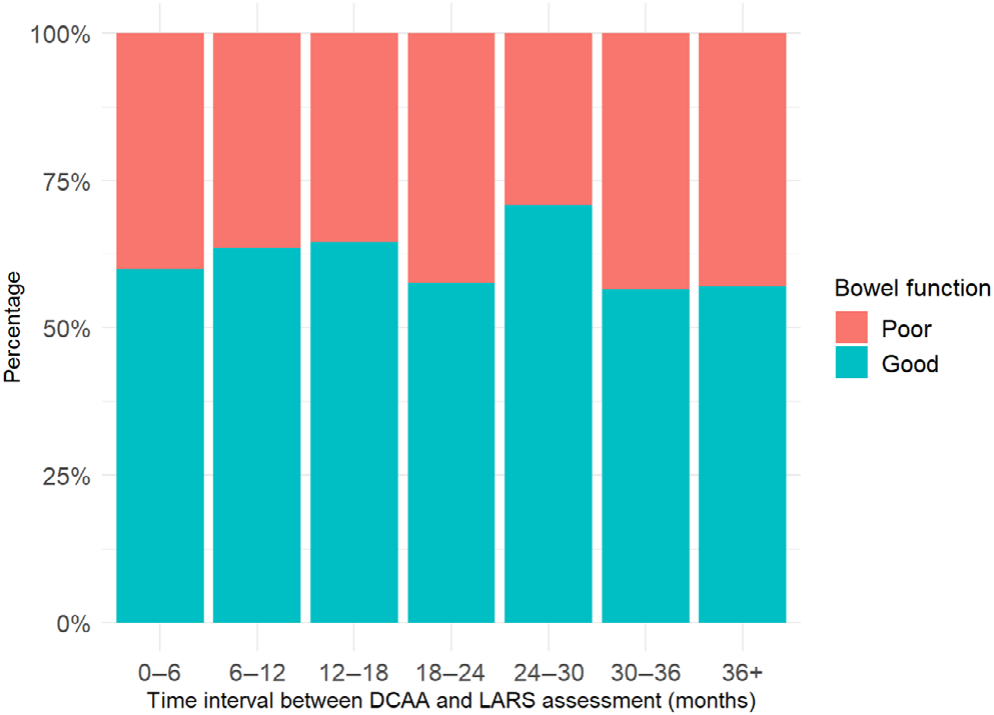
Proportion of patients with good and poor functional outcomes based on the time interval between DCAA and assessment of digestive function.

Long‐term bowel function did not depend on preoperative characteristics, including age (*P* = 0.356), the history of pelvic radiotherapy before DCAA (*P* = 0.722) or the indication for DCAA as salvage for the failure of a previous CRA or CAA (*P* = 0.755). Considering intra‐operative specifics, the bowel function obtained postoperatively was not influenced by the surgical approach (*P* = 0.197), the creation of a diverting stoma (*P* = 0.556) and the time interval between the two steps of DCAA (*P* = 0.310). Regarding postoperative variables, long‐term bowel function was not altered by the occurrence of an AL (*P* = 0.416), the overall morbidity (*P* = 0.122) or severe morbidity (*P* = 0.122). The extension of the time interval between the DCAA and the assessment of bowel function was not identified as a factor associated with a better functional outcome (Figure 2, *P* = 0.350).

In the end, the univariate analysis of all relevant preoperative, intra‐operative and postoperative factors that could have influenced bowel function after DCAA did not identify a statistically significant association in this specific context of DCAA (Table 3).

**TABLE 3:** Analysis of potential risk factors associated with a poor bowel function after DCAA.

Variables	Good bowel function	Poor bowel function	*P* value
Preoperative characteristics			
Female	58/146 (40)^a^	48/97 (49)	0.133
Age (years)	61.7 (IQR 51.4–70.8)^b^	63.0 (IQR 51.2–67.9)	0.356
BMI	24.5 (IQR 21.9–27.0)	24.8 (IQR 21.8–27.4)	0.757
ASA score ≥ 3	35/146 (24)	18/97 (19)	0.317
Active smoker	28/116 (24)	14/73 (19)	0.425
Antiplatelet therapy	18/135 (13)	10/92 (11)	0.579
Anticoagulant therapy	7/120 (5.8)	8/80 (10)	0.273
Diabetes	17/135 (13)	9/92 (9.8)	0.514
Previous pelvic radiotherapy	101/146 (69)	65/97 (67)	0.722
Primary disease			0.295
Colorectal cancer	131/146 (90)	82/97 (85)	
Endometriosis	6/146 (4.1)	7/97 (7.2)	
Gynaecological/urological malignancy	0/146 (0)	2/97 (2.1)	
Epidermoid carcinoma of the anus	1/146 (0.7)	0/97 (0)	
Others	8/146 (5.5)	6/97 (6.2)	
Surgical procedure			
Redo anastomosis (failure of previous CRA/CAA)	51/146 (35)	32/97 (33)	0.755
Redo anastomosis (failure of previous CRA/CAA) in the early postoperative period (≤21 days)	13/146 (8.9)	5/97 (5.2)	0.274
Surgical approach (first step of DCAA)			0.197
Laparotomy	78/146 (53)	45/97 (46)	
Laparoscopy	65/146 (45)	46/97 (47)	
Robot‐assisted	3/146 (2.1)	6/97 (6.2)	
Toupet procedure	17/146 (12)	6/97 (6.2)	0.155
Deloyers' manoeuvre	52/146 (36)	31/97 (32)	>0.999
Diverting stoma	52/146 (36)	31/97 (32)	0.556
Delay between two steps of DCAA (days)	8.0 (IQR 7.0–11.0)	8.0 (IQR 6.0–11.0)	0.310
Postoperative course			
Overall morbidity	78/146 (53)	42/97 (43)	0.122
Major morbidity	25/146 (17)	21/97 (22)	0.378
Intra‐abdominal colon necrosis	7/146 (4.8)	5/97 (5.2)	>0.999
Necrosis of the exteriorised colon	3/146 (2.1)	1/97 (1.0)	>0.999
Pelvic abscess after first step of DCAA	8/146 (5.5)	4/97 (4.1)	0.767
Anastomotic leakage (AL)	12/146 (8.2)	11/97 (11)	0.416
Length of stay (days)	15.0 (IQR 11.0–20.8)	14.0 (IQR 12.0–19.0)	0.646
Delay between DCAA and LARS (days)	30.1 (IQR 16.4–48.8)	33.1 (IQR 18.6–51.7)	0.350

Abbreviations: ASA score, American Society of Anesthesiologists score; BMI, body mass index; CAA, coloanal anastomosis; CRA, colorectal anastomosis; DCAA, delayed coloanal anastomosis; IQR, interquartile range; LARS, low anterior resection syndrome.

^a^ *n*/*N* (%).

^b^ Median IQR (25%–75%).

## DISCUSSION

From this multicentre cohort specifically focusing on long‐term bowel function after DCAA in 243 patients, we identified that 60% of patients achieved good bowel function without major LARS. Only 4% of patients received a stoma due to poor functional outcomes of the DCAA. None of the factors usually associated with poor functional outcomes after ICAA was identified as negatively impacting the functional result of DCAA.

Two prospective randomized studies have reported the bowel function after DCAA in the specific context of DCAA‐NoStoma as an alternative to ICAA with a diverting stoma [4, 7]. In the trial by Biondo et al. [4], the median LARS score obtained was 36.0 in the DCAA group, with no significant difference compared to the ICAA group with a diverting stoma, which had a median LARS of 30.5 (*P* = 0.45). In our cohort, the observed median LARS was 26, representing a considerable difference of 10 points lower compared to patients after DCAA in the trial. Whilst all patients in the study by Biondo et al. were evaluated at 1 year postoperatively, the timeframe was more spread out in our study. However, this time interval was not identified as associated with a modification of the functional outcome (see Figure 2), thus not explaining this difference. We did not spot any factors that could explain this considerable difference. The limited sample size (*n* = 37) analysed in the trial on functional outcomes, which was a secondary endpoint, may explain a fluctuation in the observed result. In another trial published by Evrard et al. [7], 22 patients were analysed for long‐term bowel function after DCAA‐NoStoma. The median LARS score was 25, with 41% of patients having a major LARS, which is closer to our results. In the other specific context of DCAA‐Stoma performed in a salvage situation for redo CRA or CAA, few data are available in the literature. We identified only one publication involving 26 patients reporting a median LARS of 30 in this context, also quite close to the LARS score we report [14]. This study comes from one of the centres participating in our cohort, and thus some patients are common to both works, limiting the relevance of this comparison. We did not identify other studies on the functional outcome of DCAA in the context of anastomotic salvage from other surgical teams. In our study, we observed a 4% rate only of definitive stoma due to poor functional outcomes, which is notably low. It should be noted that patient preference for opting for a stoma over poor functional outcomes is subjective and influenced by various factors, including cultural considerations. Therefore, the generalization of this result needs to be considered in the light of societal and cultural differences, among other factors, which could influence the decision‐making process between patients and surgeons.

As a reference point, major LARS exists in some people even without any rectal surgery as it has been found that this problem concerns around 15% of the general population [23, 24]. DCAA is an alternative to ICAA, and it is therefore important to ensure that this option does not expose patients to an increased risk of major LARS. One reason that has been suggested as a possible cause of worse bowel function after DCAA is the inability to create a reservoir with the obligation to perform a straight CAA in this technique [25]. The role of the reservoir remains debated to this day after CAA, but recent data challenge this hypothesis, notably the multicentre randomized controlled trial SAKK 40/04, which reports a lack of superiority of the J‐pouch or side‐to‐end anastomosis compared to the straight immediate CAA regarding bowel function [26]. Concerning the risk of LARS after low anterior resection followed by an ICAA, a population‐based study conducted in Sweden involving 481 patients reported that 53% of patients presented a major LARS after ICAA and 5% had a definitive stoma due to poor functional outcomes [27]. Another recent study reported 63% of major LARS after low anterior resection and ICAA in 132 patients [28]. Thus, we do not observe a trend towards a poorer bowel function after DCAA as we found 36% of major LARS in our study and 4% of stoma due to a poor functional result. Interestingly, a previous study demonstrated that in the specific context of redo anastomosis DCAA reported an occurrence of major LARS similar to that of the redo anastomosis through ICAA [29].

In the analysis of potential risk factors associated with a poor bowel function, we did not identify any impact of the time interval between the two steps of DCAA. This result suggests that leaving the colonic stump through the anal canal for a longer period does not impair the function of the anal sphincter, meaning that the surgeon can adjust this period without concern for the future bowel function. In our cohort, the risk of a poor bowel function was not increased in the context of redo anastomosis despite the iterative pelvic dissection with inherent difficulties in this salvage surgery and the risk of pelvic nerve injury. This result aligns with an international multicentric comparative cohort study that found no significant difference in the risk of major LARS between redo anastomosis and primary anastomosis after anterior resection [30]. In this study involving 52 redo anastomoses, 96% were ICAA and only 4% were DCAA. Thus, our analysis confirms about DCAA what was primarily known about ICAA in the context of redo anastomosis. Finally, protecting DCAA with a diverting stoma was not associated with a poorer functional outcome in our cohort. This result contradicts what has been reported after ICAA [31], namely a possible alteration in functional outcomes related to the diverting stoma, although this conclusion remains debated [32].

This work has some limitations. First, the response rate to the questionnaire by eligible patients was 63%. Nevertheless, this response rate is similar to that observed in several studies evaluating LARS in a large population [33, 34]. The absence of a control group with ICAA is a second limitation to this work, limiting the comparison to the reference technique based on literature data. Then, the LARS score was created and validated on patients who underwent surgery for rectal cancer [19], whereas 12% of our cohort did not initially undergo surgery for this condition. This could lead to modifications in the methods of functional outcome evaluation. Finally, we did not collect data on the daily treatments that patients may have taken to improve bowel function, and therefore we cannot report this information in relation to the functional outcomes obtained.

In conclusion, more than half of the patients after DCAA have a good functional outcome in the case of technical success (no chronic leakage or stoma), with less than 5% of patients subsequently receiving a stoma due to poor functional outcome. These results are at least equivalent to those reported for ICAA in the literature and indicate that use of DCAA should not be limited by concerns about a poorer functional outcome.

## AUTHOR CONTRIBUTIONS

**Maxime K. Collard:** Conceptualization; data curation; formal analysis; visualization; writing – original draft; writing – review and editing. **Jean‐Jacques Tuech:** Data curation; writing – review and editing. **Charles Sabbagh:** Data curation; writing – review and editing. **Amine Souadka:** Data curation; writing – review and editing. **Jérome Loriau:** Data curation; writing – review and editing. **Eric Rullier:** Data curation; writing – review and editing. **Frédéric Marchal:** Data curation; writing – review and editing. **Adeline Germain:** Data curation; writing – review and editing. **Stéphane Benoist:** Data curation; writing – review and editing. **Jean‐Luc Faucheron:** Data curation; writing – review and editing. **Gilles Manceau:** Data curation; writing – review and editing. **Anne Dubois:** Data curation; writing – review and editing. **Anaïs Laforest:** Data curation; writing – review and editing. **Isabelle Sourrouille:** Data curation; writing – review and editing. **Aurore Protat:** Data curation; writing – review and editing. **Diane Mège:** Data curation; writing – review and editing. **Zaher Lakkis:** Data curation; writing – review and editing. **Michel Prudhomme:** Data curation; writing – review and editing. **Simon Derieux:** Data curation; writing – review and editing. **Mehdi Ouaissi:** Data curation; writing – review and editing. **Aurélien Venara:** Data curation; writing – review and editing. **Cécile Brigand:** Data curation; writing – review and editing. **Bernard Lelong:** Data curation; writing – review and editing. **Karine Pautrat:** Data curation; writing – review and editing. **Leon Maggiori:** Data curation; writing – review and editing. **Gil Lebreton:** Data curation; writing – review and editing. **Philippe Rouanet:** Data curation; writing – review and editing. **Marc Pocard:** Data curation; writing – review and editing. **Emilie Duchalais:** Data curation; writing – review and editing. **Quentin Denost:** Data curation; writing – review and editing. **Yann Parc:** Project administration; writing – review and editing. **Jérémie H. Lefevre:** Conceptualization; formal analysis; methodology; project administration; writing – review and editing.

## FUNDING INFORMATION

No funding for this study.

## CONFLICT OF INTEREST STATEMENT

The authors have no financial disclosures or conflicts of interest to declare.

## Data Availability

The data that support the findings of this study are available from the corresponding author upon reasonable request.
